# Thermoelectric Properties of Poly(3-Hexylthiophene) Nanofiber Mat with a Large Void Fraction

**DOI:** 10.3390/ma10050468

**Published:** 2017-04-28

**Authors:** Shogo Hiura, Naoki Okada, Junma Wakui, Hikari Narita, Shinji Kanehashi, Takeshi Shimomura

**Affiliations:** Graduate School of Engineering, Tokyo University of Agriculture and Technology, Koganei, Tokyo 184-8588, Japan; sho-5.hiura@ezweb.ne.jp (S.H.); s139920s@st.go.tuat.ac.jp (N.O.); j-wakui@nichiha.co.jp (J.W.); s159562w@st.go.tuat.ac.jp (H.N.); kanehasi@cc.tuat.ac.jp (S.K.)

**Keywords:** thermoelectric property, conducting polymer nanofiber, P3HT nanofiber

## Abstract

The thermoelectric properties of a poly(3-hexylthiophene) (P3HT) nanofiber mat which has higher crystallinity—and thus exhibits larger carrier mobility—than a non-fibrous P3HT film, were investigated. No significant difference was observed in the maximum values of the power factor between the P3HT nanofiber mat and the P3HT film. However, the thermal conductivity of the nanofiber mat was less than half that of the film despite having almost the same electrical conductivity. This higher thermoelectric property of the nanofiber mat than the film is attributed to the existence of highly effective conducting pathways and a large void fraction, and the result means that the nanofiber mat was a good candidate for use as a thermoelectric material.

## 1. Introduction

The thermoelectric properties of conducting polymers have been intensively investigated for applications in lightweight, flexible, and solution-processable thermoelectric generators that can transform waste and natural heat flow into electricity. Many types of conducting polymers, such as polyaniline [1], polypyrrole [2], polyphenylvinylene [3], and polycarbazole [4], have been reported to have thermoelectric properties, with the poly(3,4-ethylenedioxythiophene) (PEDOT) family reported as having particularly high thermoelectric performance [5,6,7,8,9,10]. For example, Bubnova et al. have shown that PEDOT:tosylate (PEDOT:Tos) films have a high thermoelectric figure-of-merit *ZT* of around 0.25, which is comparable to those of inorganic thermoelectric materials [5]. Furthermore, Kim et al. have shown that PEDOT:polystyrene sulfonate (PEDOT:PSS) has an even higher *ZT* of around 0.42 [6]. Recently, Park et al. have reported PEDOT prepared from a mixture of pyridine and polyether block-copolymer with a *ZT* of around 1.02, the highest value among organic materials [7]. Among polymeric materials, the PEDOT family is known to have large conductivities but average Seebeck coefficients. In contrast, poly(3-alkylthiophene)s (P3ATs), which are also polythiophene derivatives, have large Seebeck coefficients but average conductivities. Since the *ZT* values reported for P3ATs have been around 0.03, which is an order of magnitude smaller than those of the PEDOT family [11,12,13,14], they have attracted less attention as thermoelectric materials.

Here, we need to estimate *ZT* carefully, because the transport parameters of polymer films are generally anisotropic. It has been pointed out that polymer chains in the film tend to align to the in-plain direction, so it is possible that the electric and thermal conductivities of in-plain and out-of-plain direction are quite different. Wei and co-workers have been reported to clarify the anisotropic thermoelectric property of PEDOT:PSS film [15]. P3ATs also have anisotropic alignment [16] and we need to be concerned with the anisotropy of the thermal conductivity as well as the electric conductivity.

In this study, we investigate the thermoelectric properties of the mat of a poly(3-hexylthiophene) (P3HT) nanofiber with a width and thickness of about 15 and 2 nm, respectively [17,18,19,20,21,22]. Previously, we reported that P3HT nanofiber mats have higher crystallinity, and thus exhibit larger carrier mobility, than non-fibrous pristine P3HT films [22]. Furthermore, since the nanofiber mat had a large void fraction generated by the bulky nanofiber network [23], the thermal conductivity should be decreased by this low-density structure. Therefore, P3HT nanofiber mats are expected to be good candidates for use as thermoelectric materials.

## 2. Experimental Section

### 2.1. Preparation of P3HT Nanofiber Mat

Regioregular P3HT (*M*_w_ 44,000) was purchased from Sigma-Aldrich Co., Inc. (St. Louis, MO, USA) and used without further purification. The preparation of conducting polymer nanofibers has been reported elsewhere [18,19,20]. Briefly, P3HT (25 mg) was added to an organic solvent mixture (50 g) in a flask, and 0.05 wt% P3HT solution was prepared by stirring at high temperature (>70 °C). We used a solvent mixture of anisole as a poor solvent and chloroform as a good solvent at a ratio of 3:7 v/v, which is necessary for obtaining nanofibers with high crystallinity and high mobility [20]. After the reflux was stopped, the solution was cooled gradually from 70 to 20 °C at a rate of 25 °C/h, and a change in the solution color from transparent yellow to turbid reddish brown indicated the formation of nanofibers. The nanofiber suspension was then drop-cast onto a glass-slide substrate to form a nanofiber mat. Nanofiber formation was confirmed from a topographic image obtained using scanning force microscopy (SFM) (Nanocute, Hitachi High-Tech Science Corp., Tokyo, Japan).

For comparison, a conventional non-nanofibrillar P3HT film was prepared using the following process. A 0.05 wt% P3HT solution was prepared by adding P3HT (25 mg) to a flask containing only chloroform and stirring at a high temperature (>70 °C). After the reflux was stopped, the solution was cooled gradually from 70 to 20 °C at a rate of 25 °C, and the color of the solution remained yellow. The P3HT solution was then drop-cast onto a glass-slide substrate using the same procedure for the nanofiber mat.

To measure the electrical and thermoelectric properties, doping was performed by dipping mats or films on the substrate into an acceptor solution consisting of AuCl_3_ (acceptor) dissolved in dehydrate acetonitrile (both from Aldrich Co. Inc., St. Louis, MO, USA) for 3 min. Then, mats or films were washed in pure dehydrated acetonitrile for 1 min and dried in a vacuum chamber. We varied the acceptor concentration *c* from 1.0 × 10^−2^ to 5.0 × 10^−5^ M. To measure the thermal diffusivity, doping was performed by spraying a dopant solution of 5.0 × 10^−3^ M AuCl_3_ in acetonitrile. The doping concentration was varied by changing the spraying time.

### 2.2. Characterization of Electrical Property

Two-probe electrodes were fabricated using conventional sputter deposition of Pt with a shadow mask on a glass-slide substrate. The typical gap between the electrodes *L* and the effective width of electrodes *W* were measured to be 0.5 and 1 cm, respectively, using optical microscopy. The P3HT nanofiber mat or P3HT film was fabricated by drop-casting a P3HT nanofiber suspension or P3HT solution, respectively, on the substrate with the electrodes.

The two-probe *I*–*V* measurement was performed using a source measure unit (Model 6430, Keithley Instruments, Inc., Cleveland, OH, USA) in a cryostat (CRT-006, Iwatani Industrial Gases Co., Ltd., Osaka, Japan) under vacuum below 10^−5^ Torr. The resistance was obtained from the slope of the current as a function of the applied voltage at 300 K. The electrical conductivity *σ_//_* was estimated from the measured resistance, the thickness of the mat or film measured by the SFM topographic image, *L*, and *W*. The Seebeck coefficient *S*_//_ was measured also in the cryostat. The cool side of the mat or film was chilled using a cryocompressor (CA112, Iwatani Industrial Gases Co., Ltd., Osaka, Japan), and the temperatures of the hot and cool sides were controlled with a temperature controller (DB1000, Chino Co., Ltd., Tokyo, Japan) and cryogenic temperature controller (Model 1331, Lake Shore Cryotronics Inc., Westerville, OH, USA) with a sheet heater, respectively. The induced voltage was measured using a nanovolt meter (Model 2182A, Keithley Instruments, Inc., Cleveland, OH, USA), and *S* was obtained from the slope of the induced voltage *V* as a function of the temperature difference Δ*T* from 2.0 K to 8.0 K at 300 K. All electrical properties were measured in the in-plane direction of the mat or film. The power factor (*P*_//_ = *S*_//_^2^*σ*_//_) was calculated from the corresponding values of *S*_//_ and *σ*_//_.

### 2.3. Characterization of Thermal Properties

The thermal conductivity *κ* was estimated from the thermal diffusivity *α*, the specific heat capacity *C*_p_, and the density *ρ*. A Fourier transform thermalwave analyzer (ai-Phase Mobile 1u, ai-Phase Co., Ltd., Tokyo, Japan) was used to measure *α*_⏊_ [24]. A self-standing mat or film with μm-order thickness, which was made by drop-casting the P3HT nanofiber suspension or the P3HT solution, was sandwiched on the top and bottom with heater and temperature sensors, respectively, and *α*_⏊_ was measured along the thickness direction of the mat or film. A differential scanning calorimeter (Thermos Plus DSC 8230, Rigaku Co., Ltd., Tokyo, Japan) was used to measure *C*_p_. The measurement was performed in the temperature region of 293–353 K at a heating rate of 5 K/min. To avoid melting the nanofibers, the maximum temperature was set considerably lower than the melting point of the nanofiber (around 120 °C). The value of *C*_p_ was estimated after calibration using an alumina standard. The density *ρ* of the mat was estimated from SFM topographic images as follows. The image was binarized with an appropriate threshold, and the two-dimensional proportion occupied by the nanofibers *φ* was estimated using the NIH Image software (National Institutes of Health). Then, *ρ* of the mat was calculated from this proportion and the density of P3HT film (*ρ*_0_ = 1.10 g/cm^3^ [25]) as *ρ* = *ρ*_0_*φ*^3/2^ under the assumption that *ρ* was uniform. Finally, *κ*_⏊_ of the mat or film was estimated from the product *κ*_⏊_ = *α*_⏊_*C*_p_*ρ*.

## 3. Results and Discussion

### 3.1. Electrical Properties of the P3HT Nanofiber Mat

**Figure 1:**
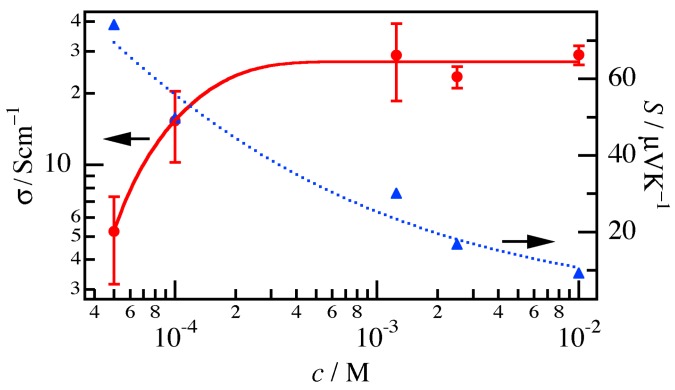
Conductivity *σ*_//_ (red circles) and Seebeck coefficient *S*_//_ (blue triangles) of the P3HT nanofiber mat treated with acceptor solutions with *c* ranging from 1.0 × 10^−2^ to 5.0 × 10^−5^ M. The solid and dotted lines are visual guides of *σ*_//_ and *S*_//_, respectively.

The conductivity *σ*_//_ and Seebeck coefficient *S*_//_ for the P3HT nanofiber mat were estimated from the *I*–*V* profiles and the *V*–Δ*T* dependence, respectively, both of which were nearly linear. Figure 1 shows the *σ*_//_ and *S*_//_ values for the nanofiber mat treated with acceptor solutions with *c* ranging from 1.0 × 10^−2^ to 5.0 × 10^−5^ M. Since the thickness of the nanofiber mats was not uniform, error bars for *σ* were estimated from the thickness at 15 different points on the sample. The results showed that *σ*_//_ increased, whereas *S*_//_ decreased with *c*, which are commonly observed tendencies in inorganic and organic semiconductors.

**Figure 2:**
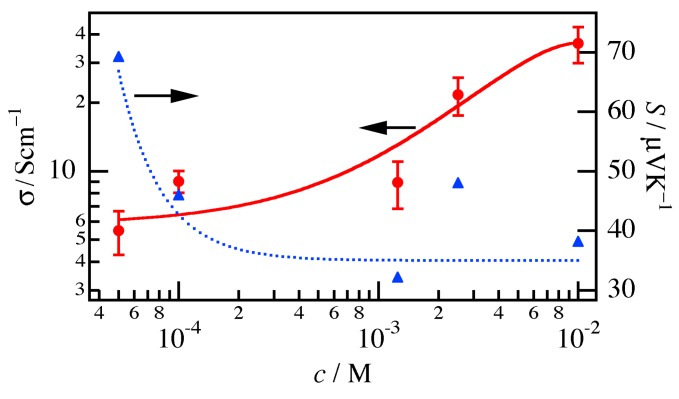
Conductivity *σ*_//_ (red circles) and Seebeck coefficient *S*_//_ (blue triangles) of the P3HT film treated with acceptor solutions with *c* ranging from 1.0 × 10^−2^ to 5.0 × 10^−5^ M. The solid and dotted lines are visual guides of *σ*_//_ and *S*_//_, respectively.

As can be seen in Figure 2, *σ*_//_ and *S*_//_ for the P3HT film exhibited a similar dependence on *c*. The error bars for *σ*_//_ were also estimated from the thickness at 15 different points on the sample. The fact that *σ*_//_ increased for higher values of *c* for the film than for the nanofiber mat (Figure 1) indicates that it took a long time to percolate the acceptor solution in the film, because the film had a denser structure than the nanofiber mat.

**Figure 3:**
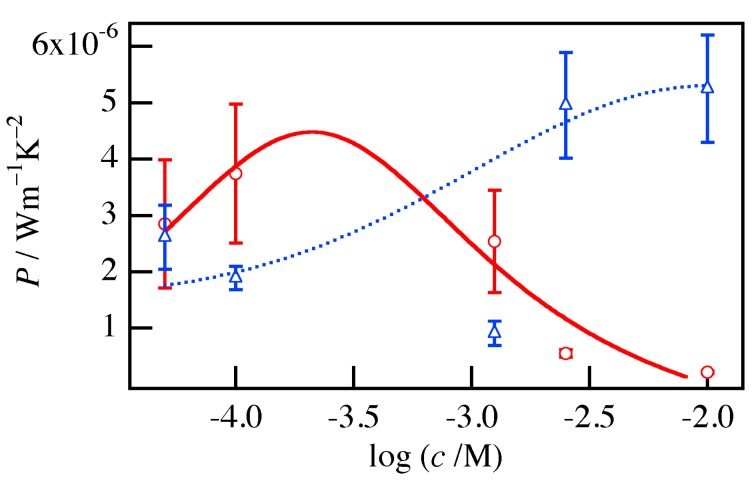
Power factor *P*_//_ of the P3HT nanofiber mat (red circles) and the P3HT film (blue triangles) treated with acceptor solutions with *c* ranging from 1.0 × 10^−2^ to 5.0 × 10^−5^ M. The solid and dotted lines show the tendencies for the mat and film, respectively.

Figure 3 shows the power factor *P*_//_ of the P3HT nanofiber mat and the P3HT film treated with acceptor solutions with *c* ranging from 1.0 × 10^−2^ to 5.0 × 10^−5^ M. Although the peak concentration of the acceptor solution was different between the mat and the film, the maximum values of *P*_//_ were of the same order, so it appears that the electrical and thermoelectric properties were similar. However, the density of the nanofiber mat was quite lower than that of the film, which means that the nanofiber has better electrical and thermoelectric properties than the film per unit weight. The superior electrical properties of nanofibers have been reported previously for field effect transistors (FETs) [20].

### 3.2. Thermal Properties of the P3HT Nanofiber Mat

**Figure 4:**
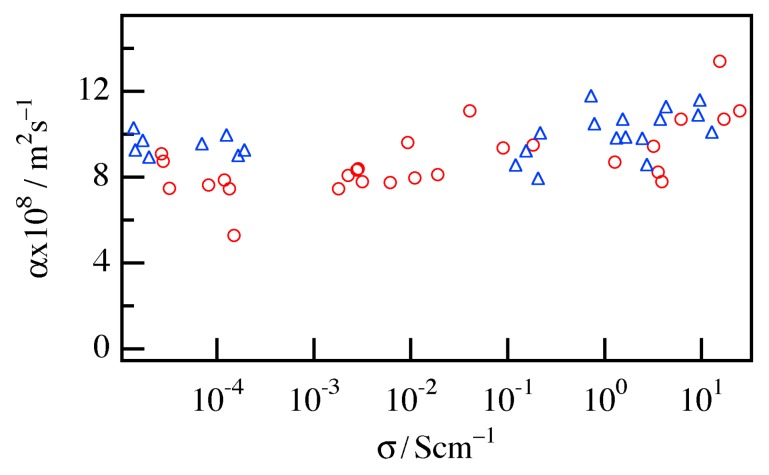
Doping concentration dependence of the thermal diffusivity *α*_⏊_ for the P3HT nanofiber mat (red circle) and the P3HT film (blue triangle).

The doping concentration dependence of the thermal diffusivity *α*_⏊_ of the mat and film are shown in Figure 4. Both profiles also indicated that *α*_⏊_ increased slightly with increasing *σ*_//_, but the increases were small and comparable to the data scatter. This behavior is attributed to the fact that the doping concentration was considerably low and that the contribution of the transport by electronic carriers, which should follow Wiedemann-Franz law, was smaller than that of the transport by the lattice phonon. From these results, we estimated *α*_⏊_ of the mat and film as 1.15 × 10^−7^ m^2^/s and 1.09 × 10^−7^ m^2^/s, respectively, as average values independent of the doping concentration. Therefore, no apparent difference between the mat and film in terms of *α*_⏊_ was observed. In addition, the specific heat capacity *C*_p_ was estimated using DSC to be 1.65 J/(g·K) and 1.32 J/(g·K) for the mat and film, respectively, and we could not also see an apparent difference between the mat and film in *C*_p_. *C*_p_ should be very weakly dependent on the doping, so these values were used in the subsequent analysis.

**Figure 5:**
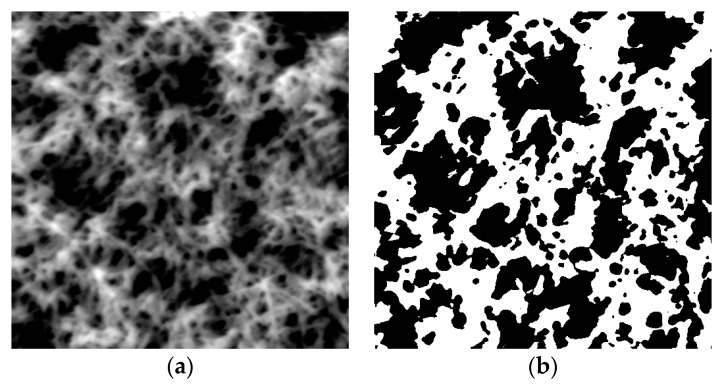
(**a**) Typical SFM topographic image of the nanofiber mat (10 × 10 μm) and (**b**) corresponding binarized image.

Figure 5 shows a typical SFM topographic image of the nanofiber mat and a corresponding binarized image. For nanofiber mats dipped in the acceptor solution with *c* below 0.1 M for 3 min, the morphology was not affected. From the binarized image, the two-dimensional proportion occupied by the nanofibers *φ* was estimated to be 0.486, and *ρ* of the mat was estimated to be 0.373 g/cm^3^. We also measured the volume of the mat and film prepared from 2.5 μL dispersion (nanofiber mat) or solution (film) both having the concentration of 0.636 g/L of P3HT. The ratio of the density between the mat and film was 0.3:1 (Figure S1), and this result was consistent with the density measured by the SFM images.

The thermal parameters of the P3HT nanofiber mat and the P3HT film are summarized in Table 1. There was no observable difference for *α*_⏊_ and *C*_p_, whereas *ρ* of the mat was less than half that of the film, so *κ*_⏊_ of the mat was extremely small. Considering the sparse structure of the nanofiber mat, the low thermal conductivity is reasonable.

### 3.3. Comparison of the Nanofiber Mat and Film

Based on the measured electrical and thermal parameters, *P*_//_*T*/*κ*_⏊_ of the P3HT nanofiber mat and the P3HT film was estimated, as shown in Table 2. The value of *P*_//_ used in the calculation was the maximum value obtained among the various values of *c* employed. *P*_//_*T*/*κ*_⏊_ of the mat was higher than that of the film. However, it should be noted that the direction (in-plane vs. out-of-plane) of the electrical quantities *σ*_//_ and *S*_//_, and of the thermal quantity *κ*_⏊_ was different. In general, the in-plane and out-of-plane values of *σ*, *S*, and *κ* for P3HT nanofiber mats as well as P3HT films are considerably different because both P3HT chains and P3HT nanofibers tend to align to the in-plane direction [15], so *ZT* estimated along the common direction of the in-plane or out-of-plane could not be obtained in this study.

It is well-known that P3HT molecules with a high regioregularity favor the edge-on alignment thermodynamically, that is, the alignment to the in-plain both in the film and nanofiber mat [26,27,28,29], so it is agreeable that *α*_⏊_ of the nanofiber mat and film were almost the same. On the other hand, *α*_//_ should be larger than *α*_⏊_ because molecules align to the in-plain direction both in the nanofiber mat and film. Here, we define the ratio of the thermal diffusivity, *α*_⏊_/*α*_//_, as *x* (<0), and consider the magnitudes of *x*_fiber_ and *x*_film_. We assumed that the thermal diffusivity was approximately decided by the lattice phonon and the electronic carriers did not relatively contribute to the thermal transport similarly to the out-of-plain measurement, the difference between *x*_fiber_ and *x*_film_ should be small, that is, *x*_fiber_ ≃ *x*_film_, because the principal direction of the thermal transport was the longitudinal direction (c-direction) of the polymer chain and both the nanofiber mat and film did not have the specific direction of polymer chains orientation in the in-plain. Using this assumption, the comparison of *P*_//_*T*/*κ*_⏊_ between the nanofiber mat and film indicates the difference of *ZT* between the nanofiber mat and film, because *ZT* corresponds to *P*_//_*T*/*κ*_//_ = *P*_//_*T*/*xκ*_⏊_.

On the other hand, the mat also had a large void fraction in the in-plane direction, and the thermal conductivity should also have the relevant difference between the mat and film in the in-plane direction.

From Table 2, the thermal conductivity of the mat was less than half that of the film because the mat had also a large void fraction in the in-plane direction, and therefore *P*_//_*T*/*κ*_⏊_ value of the mat was sufficiently smaller than that of the film. We suggest that the chief source of this advantage of the nanofiber mat can be attributed to a large void fraction decreasing the thermal conduction and the existence of highly effective pathways of the electrical conduction. So, it confirms that the nanofiber mat with a large void fraction is a good candidate for use as a thermoelectric material.

## 4. Conclusions

No significant difference was observed in the maximum values of the power factor between the P3HT nanofiber mat and the P3HT film, while the thermal conductivity of the mat was about half that of the film. These properties of the nanofiber mat were attributed to the high crystallinity, effective conducting pathways, and large void fraction. Based on our results, the *P*_//_*T*/*κ*_⏊_ values of the mat and film were calculated to be 0.016 and 0.0098, respectively, which confirms that the former is a good candidate for use as a thermoelectric material.

## Figures and Tables

**Table 1:** Thermal quantities *α*_⏊_, *C*_p_, *ρ*, and *κ*_⏊_ of the P3HT nanofiber mat and the P3HT film.

Materials	*α*_⏊_ (m^2^/s)	*C*_p_ (J/(g·K))	*ρ* (g/cm^3^)	*κ*_⏊_ (W/(m·K))
P3HT nanofiber mat	1.15 × 10^−7^	1.65	0.373	0.0708
P3HT film	1.09 × 10^−7^	1.32	1.10	0.158

**Table 2:** Power factor *P*_//_, thermal conductivity *κ*_⏊_, and thermoelectric figure-of-merit *P*_//_*T*/*κ*_⏊_ of the P3HT nanofiber mat and the P3HT film.

Materials	*P*_//_ (W/(m·K^2^))	*κ*_⏊_ (W/(m·K))	*P*_//_*T*/*κ*_⏊_
P3HT nanofiber mat	3.7 × 10^−6^	0.0708	0.016
P3HT film	5.2 × 10^−6^	0.158	0.0098

## Supplementary Materials

The following are available online at www.mdpi.com/1996-1944/10/5/468/s1.
