# Supplementary material for: Thermoelectric Properties of Poly(3-Hexylthiophene) Nanofiber Mat with a Large Void Fraction
Source: Materials (Basel). 2017 Apr 28;10(5):468. doi: 10.3390/ma10050468 (PMC5459087; doi:10.3390/ma10050468)
Supplement: Supplementary file 1 [file materials-10-00468-s001.pdf]

Supporting Information

Thermoelectric Properties of Poly(3-Hexylthiophene) Nanofiber Mat with a Large Void Fraction

Shogo Hiura, Naoki Okada, Junma Wakui, Hikari Narita, Shinji Kanehashi and Takeshi Shimomura *

[1] Graduate School of Engineering, Tokyo University of Agriculture and Technology, Koganei, Tokyo 184-8588, Japan

* Author to whom correspondence should be addressed; E-Mail: simo@cc.tuat.ac.jp; Tel.: +81-42-388-7051; Fax: +81-42-388-7051.

The density ρ of the mat was confirmed by the volume of the nanofiber mat and the film prepared from the same volume of the dispersion (nanofiber mat) or solution (film) with the same concentration. The 0.636 g/L P3HT nanofiber dispersion (in the mixture of anisole and chloroform (3:7 v/v)) and P3HT solution (in chloroform) were prepared. 2.5 μL of the dispersion or solution was dropped on the Si substrate with the thermally oxidized surface and dried under air. The foot area (the radius *R*) was measured by the optical microscope (Nikon Co. Ltd., ECLIPSE ME600), and the top height *H* was measured by the stylus type step profiler (Bruker Co. Ltd., DektakXT), then the shape was shown in Figure S1. From this result, the ratio of the density between the mat and film was 0.3:1 and this result was consistent with the density measured by the SFM images.

Figure S1. The Shape of the nanofiber mat and the film, which were made from 2.5 μL of the dispersion or solution with the same concentration of P3HT, measured by the optical microscope and the stylus type step profiler.

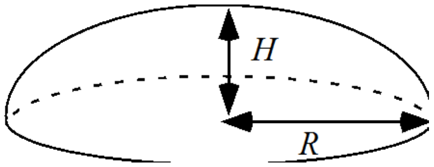

–	*R* /mm	*H* /nm
Mat	2.08	465
Film	2.35	110
